# Prone positioning during venovenous extracorporeal membrane oxygenation for acute respiratory distress syndrome: a pooled individual patient data analysis

**DOI:** 10.1186/s13054-021-03879-w

**Published:** 2022-01-06

**Authors:** Marco Giani, Emanuele Rezoagli, Christophe Guervilly, Jonathan Rilinger, Thibault Duburcq, Matthieu Petit, Laura Textoris, Bruno Garcia, Tobias Wengenmayer, Giacomo Grasselli, Antonio Pesenti, Alain Combes, Giuseppe Foti, Matthieu Schmidt, EuroPronECMO Investigators, Giacomo Bellani, Gennaro Martucci, Antonio Arcadipane, Alberto Lucchini, Eugenio Garofalo, Mirko Belliato, Vito Fanelli, Laurent Papazian, Jean-Marie Forel, Sami Hraiech, Antoine Roch, Eloi Prud’homme, Charles Edouard Luyt, Guillaume Hekimian, Juliette Chommeloux, Marc Pineton de Chambrun, Nicolas Brechot, Dawid L. Staudacher, Alexander Supady, Paul Biever, Viviane Zotzmann, Xavier Bemtgen, Asieb Sekandarzad, Kirsten Krüger, Annabelle Flügler, Erika Parmentier-Decrucq, Julien Poissy, Alexandre Gaudet, Mouhamed D. Moussa, André Vincentelli

**Affiliations:** 1 School of Medicine and Surgery, University of Milano-Bicocca, ASST Monza, Rianimazione generaleVia Pergolesi 33, 20900 Monza, Italy; 2 Department of Emergency and Intensive Care, ASST Monza, Monza, Italy; 3 Medical Intensive Care, Unit North Hospital, APHM, Marseille, France; 4 CER- eSS, Center for Studies and Research On Health Services and Quality of Life EA3279, Aix-Marseille University, Marseille, France; 5 Department of Medicine III (Interdisciplinary Medical Intensive Care), Medical Center, Faculty of Medicine, University of Freiburg, Freiburg, Germany; 6 Department of Cardiology and Angiology I, Faculty of Medicine, Heart Center Freiburg University, University of Freiburg, Hugstetterstr. 55, 79106 Freiburg, Germany; 7 Service de Médecine Intensive-Réanimation, CHU Lille, 59000 Lille, France; 8 Service de Médecine Intensive-Réanimation, Institut de Cardiologie, APHP, Sorbonne Université Hôpital Pitié– Salpêtrière, Paris, France; 9 Department of Anesthesia, Intensive Care and Emergency, Fondazione IRCCS Ca’ Granda Ospedale Maggiore Policlinico, Milan, Italy; 10 Department of Pathophysiology and Transplantation, University of Milan, Milan, Italy; 11 Sorbonne Université, INSERM, UMRS_1166-ICAN, Institute of Cardiometabolism and Nutrition, Paris, France

**Keywords:** Acute respiratory distress syndrome, Extracorporeal membrane oxygenation, Prone positioning, Pooled data analysis, Mortality

## Abstract

**Background:**

Prone positioning (PP) reduces mortality of patients with acute respiratory distress syndrome (ARDS). The potential benefit of prone positioning maneuvers during venovenous extracorporeal membrane oxygenation (ECMO) is unknown. The aim of this study was to evaluate the association between the use of prone positioning during extracorporeal support and ICU mortality in a pooled population of patients from previous European cohort studies.

**Methods:**

We performed a pooled individual patient data analysis of European cohort studies which compared patients treated with prone positioning during ECMO (Prone group) to “conventional” ECMO management (Supine group) in patients with severe ARDS.

**Results:**

889 patients from five studies were included. Unadjusted ICU mortality was 52.8% in the Supine Group and 40.8% in the Prone group. At a Cox multiple regression analysis PP during ECMO was not significantly associated with a reduction of ICU mortality (HR 0.67 95% CI: 0.42–1.06). Propensity score matching identified 227 patients in each group. ICU mortality of the matched samples was 48.0% and 39.6% for patients in the Supine and Prone group, respectively (*p* = 0.072).

**Conclusions:**

In a large population of ARDS patients receiving venovenous extracorporeal support, the use of prone positioning during ECMO was not significantly associated with reduced ICU mortality. The impact of this procedure will have to be definitively assessed by prospective randomized controlled trials.

**Supplementary Information:**

The online version contains supplementary material available at 10.1186/s13054-021-03879-w.

## Introduction

Prone positioning (PP) has proven to reduce mortality of patients with moderate to severe acute respiratory distress syndrome (ARDS) [1]. Previous studies suggested that the survival benefit of prone positioning is greater in the most hypoxemic patients [2]. Among these patients, a further worsening of respiratory failure or the impossibility of maintaining protective ventilation may require extracorporeal gas exchange, specifically venovenous extracorporeal membrane oxygenation (V-V ECMO). However, patients on V-V ECMO support have historically been managed in supine position, because of the fear of life-threatening complications associated with prone positioning during extracorporeal support and lack of experience. Recently, numerous teams have reported feasibility of PP during V-V ECMO, which might be associated with an improvement of oxygenation [3], CO_2_ clearance and respiratory system compliance [3, 4]. Several European cohort studies [3–7] evaluated the association between PP during ECMO and outcome, with conflicting results.

Two randomized controlled studies are currently undergoing in China (NCT04139733) and France (NCT04607551) to prospectively assess the impact of this procedure on duration of ECMO support and time to successful weaning from ECMO. Both studies will assess mortality as a secondary endpoint. However, since these two studies recently started patient recruitment, results will not be available soon. Until then, physicians may wonder if PP should be considered a reasonable treatment adjunct for ARDS patients on venovenous extracorporeal support. The expanding use of ECMO during the ongoing COVID-19 pandemic [8, 9] also urge to get a better knowledge of the effect of this procedure on outcomes.

We performed a pooled individual patient data analysis of five European cohort studies comparing severe ARDS patients treated with PP during V-V ECMO to “conventional” ECMO management in the supine position. The primary aim of the study was to evaluate the effect of PP during ECMO on ICU mortality.

## Methods

We pooled individual adult patient data from studies evaluating the association between the use of PP during V-V ECMO and clinical outcome, by comparing this population (Prone group) with patients managed in the supine position (Supine group). We restricted our analysis to studies published in the last 10 years (July 2011—June 2021) because of the improvements in ECMO management achieved in the last decade and the recent evidence of a proven benefit on survival of PP and ECMO in severe ARDS patients [1, 10–12].

### Search strategy

A PubMed search was done on January 15^th^ 2021 and July 31^st^ 2021 to identify cohort studies which fulfilled the eligibility criteria. “Extracorporeal membrane oxygenation” and “prone positioning” were used as key terms. Manuscripts were then reviewed to assess the eligibility. For each eligible study, we asked the corresponding author to provide fully anonymized individual patient data together with variable definitions.

### Ethics approval

All studies have been independently reviewed and approved by the local Institutional Review Boards (see Electronic Supplementary Material for approval numbers/ID).

### Data collection

We extracted from the single study databases the following parameters at baseline (i.e., before the ECMO start): age, sex, body mass index, comorbidities (see Electronic Supplementary Material for definitions), etiology of ARDS, ratio of arterial oxygen tension to inspiratory oxygen fraction (PaO_2_ to FiO_2_), Sequential Organ Failure Assessment (SOFA) score[13] at the ECMO start, and therapies before ECMO start (i.e. nitric oxide, PP, renal replacement therapy, and duration of mechanical ventilation). The ECMO configuration was described according to the ELSO Maastricht treaty [14]. Complications of PP maneuvers were recorded. We also collected duration of ECMO support, ICU length of stay and outcome at hospital discharge.

For patients who underwent PP during ECMO, we also collected the ECMO day when PP was started, and the number of PP cycles.

### Endpoints

The primary endpoint was to assess the association between the use of PP during ECMO and ICU mortality in patients with ARDS. The secondary endpoint included the assessment of the association of PP during ECMO with hospital mortality, rate of successful ECMO weaning, ICU length of stay and duration of time to successful ECMO weaning within 60 days from ECMO start. Moreover, as an explorative endpoint, we aimed to assess if the use of PP during ECMO was associated with improved outcome in specific pre-defined patient subgroups.

All studies have been independently reviewed and approved by each local Institutional Review Boards.

### Statistical analysis

Continuous data were reported as mean ± standard deviation or by median (interquartile range), according to the data distribution. Data normality was assessed by Shapiro–Wilk test and by visual inspection using histograms of distribution. Categorical data were reported as count (proportion). There was no imputation for missing data. To assess differences between patients who underwent prone positioning during venovenous ECMO (Prone group) or who were managed in supine position (Supine group) we performed unpaired Student’s T-test (or Wilcoxon–Mann–Whitney test) and Chi-square test (or Fisher’s exact test) in continuous and categorical variables, respectively.

ICU survival was described between Prone and Supine group by the Kaplan–Meier approach. The date of hospital discharge (or death after ICU discharge) was not available in all patients. For this reason, patients discharged alive from ICU before 60-days were right-censored. Statistical difference between the survival curves was assessed by log-rank test.

We explored the independent association with 60-day ICU mortality by a COX-proportional regression model. We included in this model a set of clinically meaningful variables which were selected a-priori: age; sex; obesity; chronic respiratory disease, chronic heart failure, malignancy, immunodeficiency, chronic liver disease; PaO_2_/FiO_2_; SOFA score; mobile ECMO transfer; duration mechanical ventilation days before ECMO; use of PP before ECMO and use of PP during ECMO. The comorbidities included in the model were chosen based on previous literature suggesting their independent role on outcome in critically ill patients with ARDS [15, 16]. Independent predictors of hospital mortality were assessed by a multiple logistic regression model using the same abovementioned clinically meaningful variables.

Results of multivariable models were reported as odds ratio (OR) or hazard ratio (HR) with 95% confidence interval (95% CI) for logistic and COX-proportional regression models, respectively, all adjusted for robust clustering by using the original study as cluster variable.

The propensity score matching method was applied to estimate the effect of PP during ECMO on main outcomes. Details on matching technique are provided in the Supplemental Methods (see Electronic Supplementary Material). Difference in 60-day survival probability was estimated between matched Prone and Supine groups by the Kaplan–Meier approach and compared by the log-rank test. For the matched cohorts we also described ICU and hospital mortality, rate of successful ECMO weaning, ICU length of stay and duration of time to successful ECMO weaning within 60 days from ECMO start [4].

To explore the potential role of prone positioning in specific subgroups of ECMO patients, we evaluated the association between Prone and Supine groups with hospital mortality in pre-defined clinically relevant subgroups. Patients were then classified according to age (age > 50); obesity (cutoff: BMI > 30 kg/m^2^); SOFA score (SOFA > 12); baseline PaO2/FiO2 > 60; use of prone position before ECMO; duration of mechanical ventilation before ECMO (> to 7 days).

Statistical significance was reported with a p-value < 0.05 (two-sided). Statistical analyses were performed using STATA/MP 16.0 for Windows (StataCorp LLC, College Statio, TX 77,845, USA) and GraphPad Prism 8 for Windows (version 8.0.2, GraphPad Software, Inc.).

## Results

From the 151 references identified by the search strategy, five manuscripts fulfilled the eligibility criteria and were included in the analysis (see Table 1).

**Table 1:** Characteristics of the studies included in the analysis

Study	Guervilly et al. [6]	Garcia et al. [5]	Rilinger et al. [7]	Giani et al. [3]	Petit et al. [4]
Design	Monocentric retrospective cohort study	Monocentric retrospective cohort study	Monocentric retrospective cohort study	Multicentric retrospective cohort study	Monocentric retrospective cohort study
Country	France	France	Germany	Italy	France
Case volume^#^ of the ECMO centers, runs/year (V-V runs)	55 (50)	45 (25)	125 (35)	35–55 (25–33)^&^	450 (60)
Total number of patients (Prone group)	168 (91)	25 (14)	158 (38)	240 (107)	298 (64)
Population	ARDS patients on	COVID-19 ARDS patients on V-V ECMO	ARDS patients on V-V ECMO	ARDS patients on V-V ECMO	ARDS patients on V-V ECMO
Prone positioning duration	16 (12–16) hours	16 (15–17) hours	20 (17–21) hours	15 (12–18) hours	16 (16–16) hours
Statistics	Univariable analysis, individual matching	Univariable analysis	Multivariable analysis, propensity score matching	Multivariable analysis, propensity score matching	Propensity score matching
Outcome					
Last follow-up	90 days	28 days	Hospital discharge	Hospital discharge	90 days
Mortality, Prone vs Supine group	36% vs 58%*	79% vs 27%°	63% vs 63%*	30% vs 53%*	25% vs 46%*

^&^Annual case volume before the COVID-19 pandemic

^%^Case volume range of the centers participating to the multicentric study

^*^Mortality rates of matched patient cohorts

°Unadjusted mortality

Unadjusted ICU and hospital mortality of the 5 included study are presented as a Forest plot graph in the see Additional file 1 (Fig. E1).

A total of 889 patients were included, 575 in the Supine group and 314 in the Prone group. The more frequent ECMO configuration was the femoro-jugular approach (66%), followed by femoro-femoral (18%) and jugular dual-lumen (16%) cannulation.

Patients in the Prone group underwent a median of 2 (1–3) PP session while on V-V ECMO, for a total of 824 PP sessions during ECMO support. Median ECMO duration before the first prone positioning was 5 (2–6) days. Complications were reported in 53 (6.4%) PP sessions. 9 (1.1%) PP cycles were aborted due to complications. We did not record any accidental extubation or dislodgement of the ECMO cannulae. One patient suffered an episode of ventricular arrhythmia while on prone position. Bleeding from cannula insertion site and drop of the extracorporeal blood flow occurred in 16 (1.9%) and 15 (1.8%) cases, respectively, and were the most frequent complications. Other reported complications were vomiting (13, 1.6%) and hemodynamic instability (8, 1%).

Table 2 describes the study population according to the study group. Age, PaO2 to FiO2 ratio and duration of mechanical ventilation before ECMO were similar in the two study groups. Median BMI was higher in the Prone group. Immunodeficiency was more frequent in the Supine group, whereas the SOFA score was higher compared to the Prone group. PP before ECMO was used more frequently in the Prone group.

Unadjusted ICU, 60 days (see Kaplan–Meier analysis, Fig. E2, Additional file 1) and hospital mortality were lower in the Prone group.

Independent predictors of 60-day ICU mortality are displayed in Table E1 (see Additional file 1). Malignancy, chronic liver disease and SOFA score were independently associated with higher mortality. The reduction of ICU mortality in patients who underwent PP during ECMO was not statistically significant (HR 0.67 95% IC: 0.42–1.06).

Predictors of hospital mortality are displayed in Table E2 (see Additional file 1). Age, chronic heart failure, malignancy, immunodeficiency, chronic liver disease and SOFA score were independently associated with higher hospital mortality. The use of PP during ECMO was not significantly associated with mortality reduction (HR 0.79 95% IC: 0.46–1.35).

A total of 227 propensity score-matched patients were identified in each group. Characteristics and outcomes of the matched populations are provided in Table 3. In the “matched” Prone group, patients underwent a median of 2 (1–3) prone positioning maneuvers, which started after 5 (2–5) days of ECMO support.

**Table 2:** Patient baseline characteristics, pre-ECMO parameters and outcomes in the Supine and Prone group

Baseline characteristics	Supine group (n = 575)	Prone group (n = 314)	p-value
Age, years	52 (41–61)	51 (39–61)	0.506
Sex, female	191 (33.2)	91 (29.0)	0.195
BMI, kg/m^2^	27 (23–32)	29 (25–34)	0.003
Pre-existing conditions at baseline			
Obesity (BMI ≥ 30)	180 (35.7)	113 (42.3)	0.072
Hypertension	105 (32.1)	58 (23.9)	0.031
Diabetes	88 (15.7)	53 (17.3)	0.547
Chronic respiratory disease	94 (16.8)	37 (12.1)	0.064
Malignancy	68 (12.1)	28 (9.1)	0.178
Vascular disease	57 (10.2)	27 (8.8)	0.515
Chronic heart failure	54 (9.6)	30 (9.8)	0.944
Chronic renal disease	22 (3.9)	17 (5.5)	0.272
Chronic liver disease	34 (6.1)	10 (3.2)	0.072
Immunodeficiency	101 (18.0)	36 (11.7)	0.015
ARDS etiology			< 0.001
Pulmonary ARDS	405 (70.4)	234 (74.5)	
Extrapulmonary ARDS	58 (10.1)	52 (16.6)	
Other/unknown	112 (19.5)	28 (8.9)	
COVID-19 ARDS	11 (1.9)	14 (4.5)	0.028
Mobile ECMO transfer	336 (59.5)	215 (68.5)	0.008
Pre-ECMO variables			
PaO_2_/FiO_2_, mmHg	67 (56–80)	66 (55–80)	0.568
SOFA score	12 (9–16)	10 (8–13)	< 0.001
Use of nitric oxide	189 (33.3)	91 (29.0)	0.184
Use of prone positioning	248 (45.1)	180 (57.3)	0.001
Use of renal replacement therapy	61 (13.7)	31 (11.3)	0.362
Days of mechanical ventilation before ECMO	3 (1–8)	3 (1–7)	0.632
Outcomes			
ECMO duration, days	8 (4–17)	15 (10–26)	< 0.001
ECMO successful weaning	326 (57.0)	211 (67.2)	0.003
ICU LOS, days	20 (10–36)	31 (19–48)	< 0.001
Survivors, n = 449	27 (15–45)	34 (23–49)	< 0.001
Non-survivors, n = 397	13 (4–26)	23 (16–41)	< 0.001
Time to successful ECMO weaning within 60 days from ECMO start, days	60 (8–60)	28 (12–60)	0.906
ICU mortality, n (%)	298 (52.8)	128 (40.8)	0.001
Hospital mortality, n (%)	306 (54.4)	136 (43.5)	0.002

Data are presented as count (percentage) or median (25th-75th percentile). BMI, body mass index; LOS, length of stay

**Table 3:** Characteristics and clinical outcomes of matched sample of patients in the Supine vs Prone group

Variable	Supine group, n = 227	Prone group, n = 227	Standardized difference
*Baseline characteristics*			
Age, years	53 (40–62)	51 (40–61)	− 0.08
Sex, males	158 (69.6)	159 (70.0)	0.01
Obesity (BMI ≥ 30)	93 (41.0)	93 (41.0)	0
Chronic respiratory disease	30 (13.2)	27 (11.9)	− 0.04
Chronic heart failure	21 (9.3)	21 (9.3)	0
Chronic liver disease	6 (2.6)	8 (3.5)	0.05
Malignancy	24 (10.6)	25 (11.0)	0.01
Immunodeficiency	35 (15.4)	34 (15.0)	− 0.01
*Clinical illness severity*			
SOFA score	11 (8–14)	11 (8–14)	0
Baseline PaO_2_/FiO_2_, mmHg	67 (56–77)	66 (55–80)	− 0.01
*Before ECMO*			
Days of mechanical ventilation before ECMO	4 (1–8)	3 (1–8)	− 0.03
Prone positioning before ECMO	126 (55.5)	124 (54.6)	− 0.02
Mobile ECMO transfer	152 (67.0)	150 (66.1)	− 0.02
			*p-value*
*Outcome variables*			
ECMO duration, days	9 (5–25)	15 (10–25)	< 0.001
ECMO successful weaning	135 (59.5)	148 (65.2)	0.208
ICU LOS, days	24 (12–41)	31 (19–48)	< 0.001
Survivors	27 (15–49)	34 (22–49)	0.024
Non-survivors	19 (7–33)	23 (15–47)	0.005
Time to successful ECMO weaning within 60 days from ECMO start, days	56 (8–60)	24 (12–60)	0.705
ICU mortality	109 (48.0)	90 (39.6)	0.072
Hospital mortality	113/226 (50.0)	94/226 (41.6)	0.073

Data are presented as count (percentage) or median (25th-75th percentile). SOFA, Simplified Organ Failure Assessment; LOS, length of stay

ECMO duration was significantly lower in the Supine group, whereas both ICU and hospital survival rates were 8.4% higher in the Prone group (*p* = 0.072 and 0.073, respectively).

**Fig.1:**
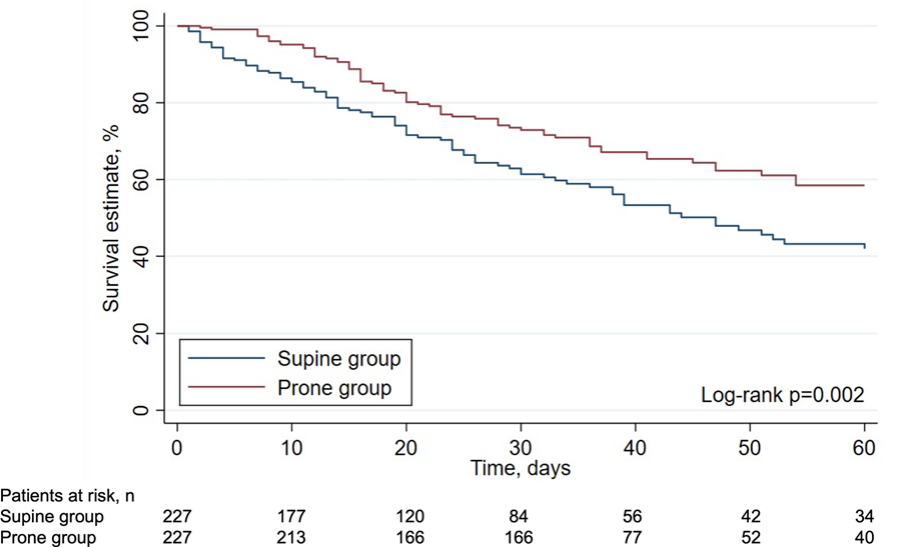
Survival estimation over 60-day follow-up in matched groups of patients. For each time interval, the survival probability was calculated as the number of alive subjects divided by the number of patients at risk

Kaplan–Meier survival analysis showed a lower 60-day mortality in the Prone group (log-rank test *p* = 0.002, see Fig. 1).

**Fig.2:**
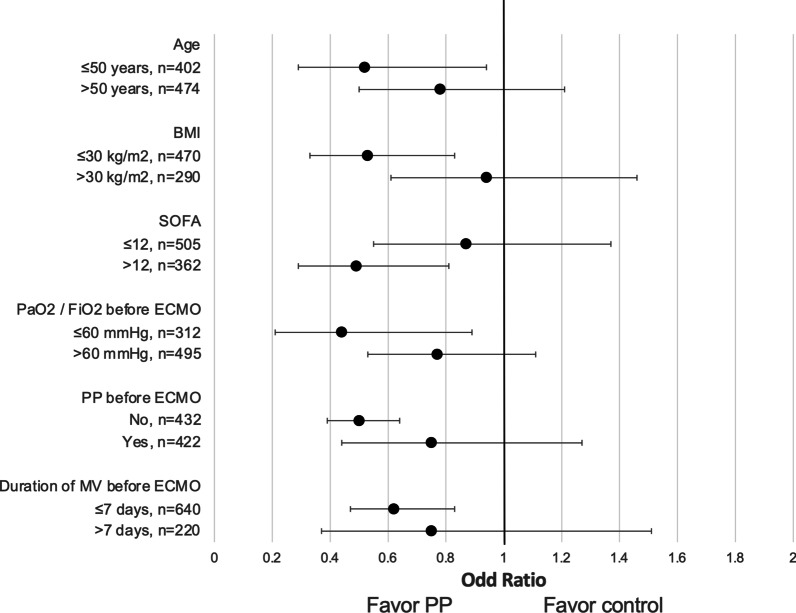
Post-hoc analysis on risk of hospital mortality of patients who underwent prone positioning (PP) during ECMO versus patients managed in supine position (control) in predefined clinically relevant subgroups. Risk of hospital mortality was expressed using OR (points) with 95% CI (error bars) adjusted by robust clustering taking into account the 5 original cohorts of ECMO patients (i.e. clusters). BMI, Body Mass Index; SOFA, Sequential Organ Failure Assessment; MV, mechanical ventilation

As an exploratory endpoint, we assessed the association between the use of PP during ECMO and hospital mortality in pre-defined clinically relevant subgroups (Fig. 2). The strongest association between PP during ECMO and decrease of hospital mortality was found in patients ≤ 50 years of age, BMI ≤ 30 kg/m^2^, SOFA > 12, PaO2 to FiO2 ratio ≤ 60 mmHg, in patients who were not proned PP before ECMO and in those with a duration of mechanical ventilation before ECMO ≤ 7 days.

## Discussion

In this pooled individual patient data analysis of European cohort studies including 889 patients, the use of prone positioning during ECMO was not independently associated with a significant reduction of ICU mortality. Although matched patients in the Prone group had an absolute decrease of ICU mortality of 8.4% (39.6% vs 48%), the difference in mortality did not reach statistical significance (*p* = 0.072). However, when patients were matched on baseline characteristics using a propensity score, those in the Prone group had a lower 60-day mortality.

Low volume-low pressure ventilation and repeated and prolonged prone positioning proved to decrease mortality in moderate to severe ARDS patients [1].

Although mechanical ventilation during ECMO [17] and other aspect of V-V ECMO management vary significantly among centers [18], very low tidal volume (< 4 ml/kg/predicted weight) and driving pressure are usually utilized to minimize the risk of ventilator induced lung injury.

The application of ultraprotective ventilation after V-V ECMO implantation may lead to derecruitment and decrease of respiratory system compliance [19]. PP may contribute to maintain lung recruitment while avoiding high airway pressures [20]. Previous studies [2] showed that the highest benefit of PP on survival is observed in the most severe patients. Therefore, it seems reasonable to investigate the efficacy of PP in patients supported by V-V ECMO.

To date, despite a sound physiologic rationale, the quality of evidence supporting the use of PP during ECMO is low and derives only from retrospective cohort studies. These studies gave conflicting results, since three of them [3, 4, 6] showed an independent association between the use of PP during extracorporeal support and reduced 90-day or hospital mortality, whereas the fourth one failed to show a beneficial effect of PP during ECMO on patient outcome. Another small cohort study [5] found an increased mortality of ECMO patients who underwent PP, which however was used only as rescue therapy for refractory hypoxia. Of note, this was the only study on patients with COVID-19 associated ARDS, in whom the efficacy of PP remains to be demonstrated [21]. COVID-19-related ARDS has a different pathophysiology than “traditional” ARDS, resulting in an ARDS phenotype with lower lung elastance and less recruitability[22]. Therefore, the benefit of PP on mortality might not be translated to COVID-19 ARDS.

Recently, the findings of the available studies on the use of PP during ECMO were pooled in in a systematic review and meta-analysis [23] that showed a non-significant trend towards decreased mortality in patients who underwent PP during ECMO. However, this meta-analysis by Poon et al. (which also included 4 out of 5 studies included in our analysis) was performed on aggregate data (i.e. the authors did not have access to patient data), and may be biased by an imbalance between the characteristics of patients treated with PP and controls. Our analysis, on the contrary, was performed on individual patient data, thus allowing to adjust for confounders. Individual patient data analysis may allow to homogenize and to minimize bias, while increasing the sample size and, consequently, the statistical power [12]. Due to the nature of the studies included in our analysis, our findings cannot support the routine use of PP. Nonetheless, the trend towards reduced mortality suggests that it is very unlikely that PP during V-V ECMO is associated with harm. Indeed, our study reports a low incidence of adverse events related to this procedure. However, it should be noted that PP during ECMO is a challenging procedure, and might be associated with more complications when performed in ECMO centers without a specific expertise. Moreover, PP is time-consuming and might be difficult to perform as requires four to six health care providers. This might be challenging especially in the COVID-era. For these reasons, we speculate that PP may be considered in experienced centers for the most severe ECMO patients, who present with a high degree of consolidation and lung collapse. Moreover, we might hypothesize that anticipating the commencement and increasing the number of PP procedures during ECMO (median of 2 (1–3) procedures in our study versus an average of 4 ± 4 in the PROSEVA trial [1]) might provide a greater benefit on survival.

In an exploratory analysis, we found a stronger association between PP and lower hospital mortality in patients who were younger (≤ 50 years of age), more severe (SOFA > 12, PaO2 to FiO2 ratio ≤ 60 mmHg), in patients who were not proned PP before ECMO and in those with a shorter duration of mechanical ventilation before ECMO (less than ≤ 7 days). This might indicate a greater benefit of PP in the most acute and severe patients, who have higher mortality. In addition, BMI over 30 was not associated with beneficial effects of PP. We hypothesize that ECMO patients with BMI < 30 may have sicker lungs, and thus may benefit more from PP compared to obese patients, where severe hypoxia may be partly secondary to airway or lung collapse due to high pleural pressure. Therefore, the risk/benefit ratio of proning in this subgroup should be carefully evaluated.

This study presents several limitations. First, only baseline variables were used for statistical corrections. Multiple regression and propensity score analysis do not take into account several other unmeasured variables. Data were aggregated from five studies which were not randomized trials. Therefore, we cannot exclude that clinicians decided to use PP in specific subsets of patients (i.e. selection bias). However, we may expect that clinicians tend to use PP in patients who do not improve or even transiently worsen after ECMO connection, whereas a significant improvement in oxygenation after the ECMO start might suggest keeping the patient in the supine position. Hence, this potential bias might play “against” PP in our analysis. This might explain the longer ECMO support in the Prone group, even if we cannot exclude that a longer duration of ECMO might be due to a “selection” of patients for which clinicians want to struggle more. Second, the studies included in the analysis were not homogeneous: only one study [3] was multi-centric and gathered control patients from two large “non-pronating” ECMO centers in order to minimize selection bias. Third, our search cannot be considered systematic, as we did not include all the small studies and case series who reported the use of PP in ECMO patients without comparing outcomes to (matched or unmatched) controls. Fourth, date of hospital discharge (or date of death after ICU discharge) was not available. For this reason, we could not perform a time-to-event analysis on hospital survival. Fifth, when gathering data on complications, we did not collect information on pressure sores and other skin lesions attributable to PP. Last, we did not gather physiologic data before and after prone positioning. Therefore, we did not assess if variation of oxygenation, CO_2_ clearance and respiratory system compliance are associated with patient outcome.

## Conclusion

In our pooled individual patient data analysis, the use of prone positioning during ECMO was not associated with reduced mortality. Ongoing prospective randomized controlled trials will have to assess the impact of this procedure on mortality and other patient-centered outcomes.

## Supplementary Information

**Additional file 1.** Supplemental methods and results.

## Data Availability

The datasets used and/or analyzed during the current study are available from the corresponding author on reasonable request.

## Abbreviations

PP: Prone positioning
ARDS: Acute respiratory distress syndrome
ECMO: Extracorporeal membrane oxygenation
V-V: Veno-venous
ICU: Intensive care unit
SOFA: Sequential organ failure assessment
PaO2: Arterial oxygen tension
FiO2: Inspiratory oxygen fraction
MV: Mechanical ventilation
BMI: Body mass index
LOS: Length of stay
OR: Odds ratio
HR: Hazard ratio
CI: Confidence interval
