# Supplementary material for: Prone positioning during venovenous extracorporeal membrane oxygenation for acute respiratory distress syndrome: a pooled individual patient data analysis
Source: Crit Care. 2022 Jan 6;26:8. doi: 10.1186/s13054-021-03879-w (PMC8731201; doi:10.1186/s13054-021-03879-w)
Supplement: Supplementary file 1 — Additional file 1. Supplemental methods and results. [file 13054_2021_3879_MOESM1_ESM.docx]

**Electronic Supplementary Material**

**Prone Positioning during venovenous Extracorporeal Membrane Oxygenation for Acute Respiratory Distress Syndrome: a Pooled Individual Patient Data Analysis**

Marco Giani^,^ Emanuele Rezoagli, Christophe Guervilly, Jonathan Rilinger, Thibault Duburcq, Matthieu Petit, Laura Textoris, Bruno Garcia, Tobias Wengenmayer, Antonio Pesenti, Giacomo Grasselli, Alain Combes, Giuseppe Foti and Matthieu Schmidt for the EuroPronECMO Investigators

- Corresponding Author

Dr. Marco Giani, MD

University of Milano-Bicocca, Rianimazione generale, ASST Monza, Italy

[marco.giani@unimib.it](mailto:marco.giani@unimib.it)

**Supplemental Methods**

*Ethics approval*

All studies have been independently reviewed and approved by the local Institutional Review Boards. Here we provide the approval number / ID for each study.

- Giani et al. ref. 3105
- Petit et al. no. 2217028v0
- Guervilly et al. CIL/APHM 2018-44
- Rilinger et al. EK-Freiburg 151/14
- Garcia et al. ID-CRB 2020-A00763-36

*Comorbidities definitions*

Chronic respiratory disease includes either COPD or asthma; Malignancy includes either solid or hematological proliferative disorder; Vascular disease includes coronary or systemic vascular disease; immunodeficiency includes primary immunodeficiency, autoimmune disorder, and immunosuppressive therapy. Pulmonary ARDS includes pneumonia and trauma. Extrapulmonary ARDS includes sepsis/septic shock.

*Propensity score*

Patients were matched using the nearest neighbor approach (1:1 matching with no replacement) using a caliper of 0.2 standard deviation of the logit of the propensity score.

The similarity of the matched groups was assessed by the standardized differences of each independent variable used in the propensity score estimation. The balance in measured variable among the 2 groups (i.e. prone versus supine group) was investigated by standardized difference. A threshold of 0.10 of standardized difference was used as a cut-off for negligible balance

Statistical significance in the difference between the groups on continuous (i.e. ICU length of stay and the time to successful ECMO weaning within 60 days from ECMO start) and categorical outcome variables (i.e. ICU and hospital mortality, and disconnection from ECMO) were ascertained by Wilcoxon rank-sum test and Chi-square test, respectively.

**Supplemental Results**

**Figure E1**


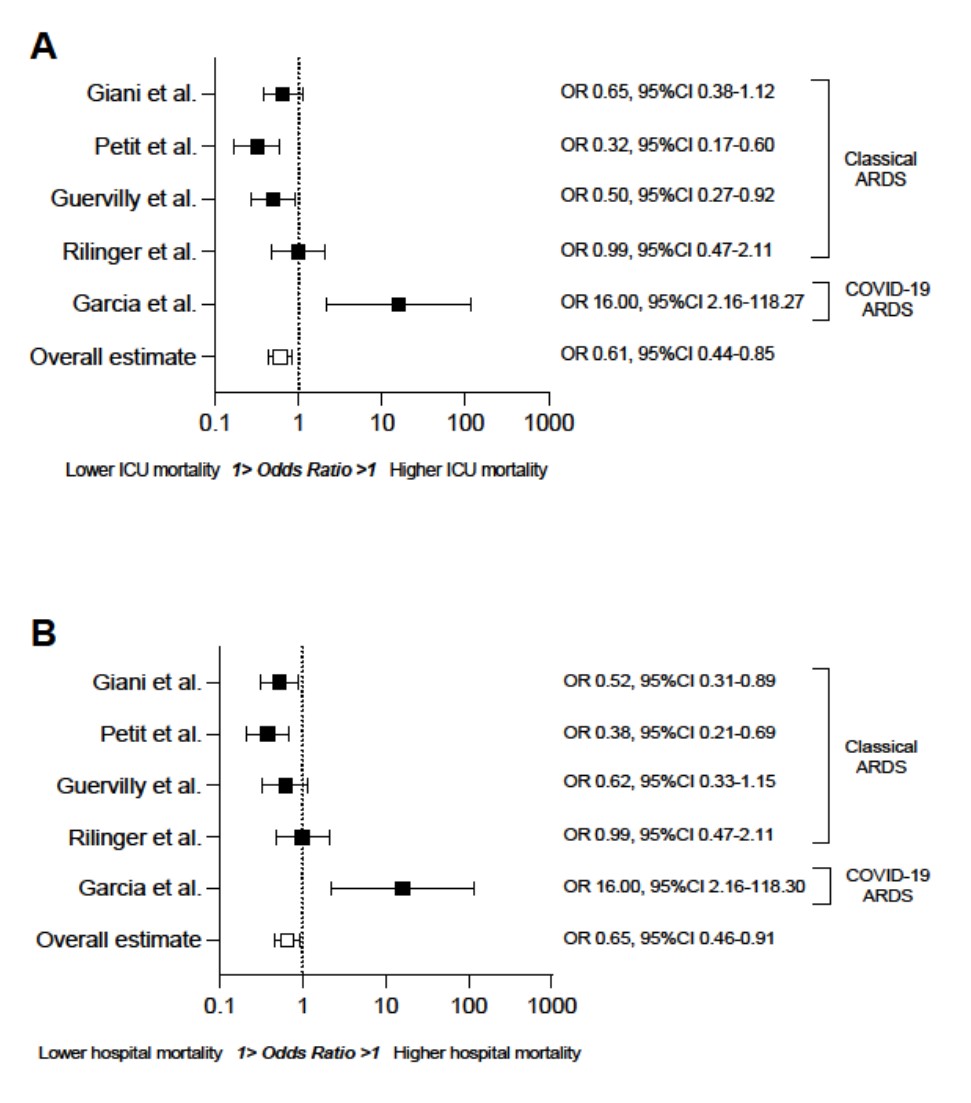


**Odds ratio with 95% CI of ICU (panel A) and hospital (panel B) mortality in ECMO patients who underwent prone positioning (vs supine patients) in the 5 included studies.** The overall data represents the Odds ratio with 95% CI of mortality by merging all data from the 5 studies and 95% CI was adjusted using the type of study as cluster variable by robust clustering.

**Figure E2.**


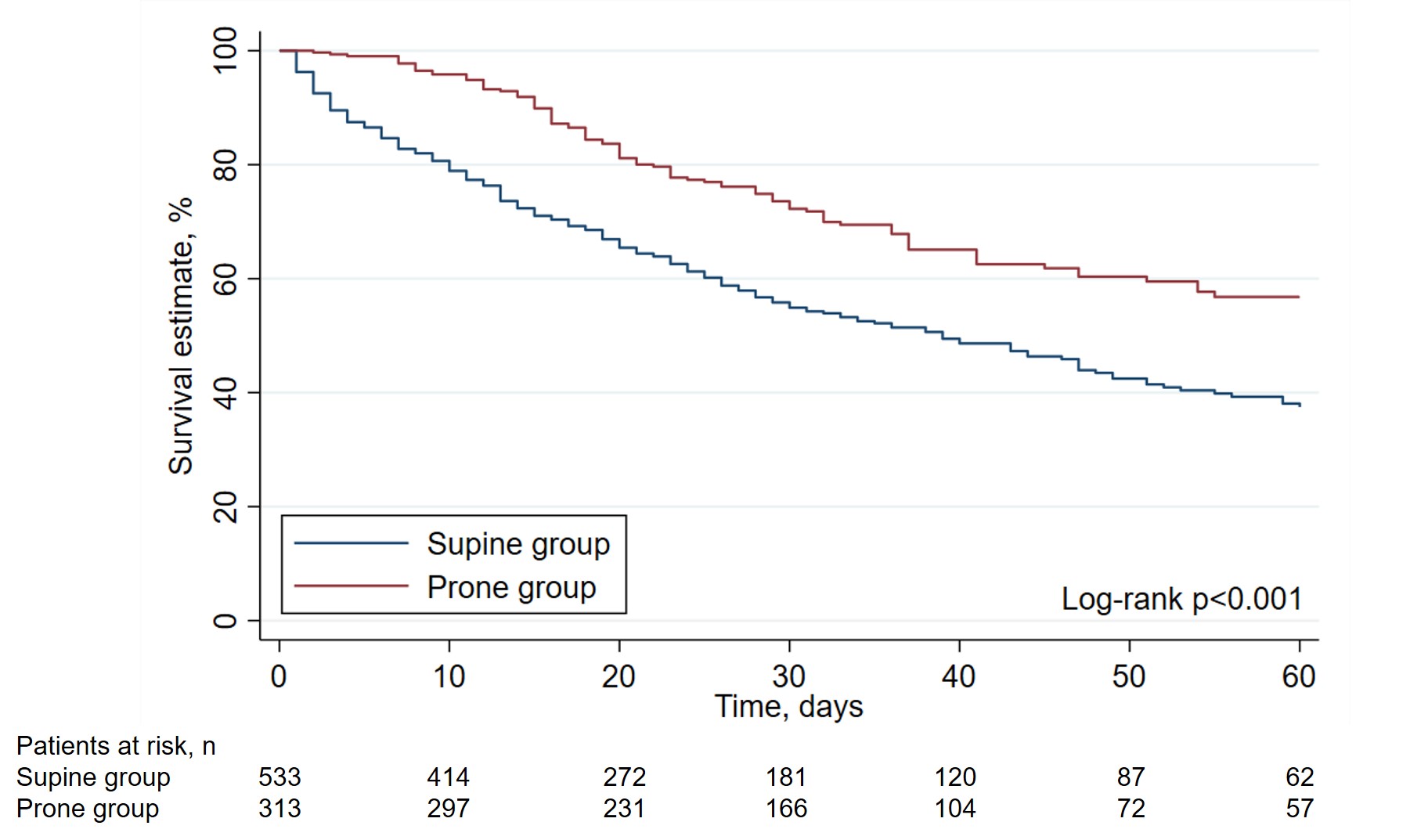


**Survival estimation over 60-day follow up stratified by prone positioning during ECMO.**

**Table E1**

| **Variable** | **HR** | **95% CI** | **p** |
| --- | --- | --- | --- |
| Age, years | 1.01 | 1.00-1.02 | 0.117 |
| Sex, male | 0.97 | 0.84-1.11 | 0.632 |
| Obesity (BMI≥30) | 0.94 | 0.77-1.16 | 0.576 |
| Chronic respiratory disease | 1.38 | 0.87-2.18 | 0.168 |
| Chronic heart failure | 0.91 | 0.65-1.27 | 0.589 |
| Chronic liver disease | 2.24 | 1.55-3.25 | <0.001 |
| Malignancy | 2.22 | 1.89-2.61 | <0.001 |
| Immunodeficiency | 1.03 | 0.89-1.20 | 0.676 |
| SOFA | 1.11 | 1.06-1.15 | <0.001 |
| PaO_2_/FiO_2_ before ECMO, mmHg | 1.00 | 1.00-1.01 | 0.622 |
| Days of mechanical ventilation before ECMO | 1.00 | 0.99-1.01 | 0.882 |
| Use of prone positioning before ECMO | 0.87 | 0.67-1.12 | 0.281 |
| Mobile ECMO transfer | 0.53 | 0.40-0.70 | <0.001 |
| Use of prone positioning during ECMO | 0.67 | 0.42-1.06 | 0.090 |

**Predictors of ICU mortality at 60-day follow-up.** 95% CI of the HR was adjusted by robust clustering taking into account the 5 original cohorts of ECMO patients (i.e. clusters).

**Table E2.**

| **Variable** | **OR** | **95% CI** | **p** |
| --- | --- | --- | --- |
| Age, years | 1.02 | 1.01-1.03 | 0.001 |
| Sex, males | 1.04 | 0.81-1.33 | 0.777 |
| Obesity (BMI≥30) | 0.66 | 0.41-1.05 | 0.079 |
| Chronic respiratory disease | 1.43 | 0.83-2.44 | 0.196 |
| Chronic heart failure | 0.63 | 0.42-0.95 | 0.026 |
| Chronic liver disease | 3.37 | 1.83-6.19 | <0.001 |
| Malignancy | 3.17 | 2.27-4.41 | <0.001 |
| Immunodeficiency | 1.40 | 1.18-1.66 | <0.001 |
| SOFA | 1.08 | 1.03-1.14 | 0.002 |
| Baseline PaO_2_/FiO_2_, mmHg | 1.00 | 1.00-1.01 | 0.477 |
| Days of mechanical ventilation before ECMO | 1.02 | 1.00-1.03 | 0.052 |
| Use of prone positioning before ECMO | 1.21 | 0.85-1.71 | 0.285 |
| Mobile ECMO transfer | 0.65 | 0.50-0.86 | 0.002 |
| Use of prone positioning during ECMO | 0.79 | 0.46-1.35 | 0.385 |

**Predictors of hospital mortality.** 95% CI of the OR was adjusted by robust clustering taking into account the 5 original cohorts of ECMO patients (i.e. clusters).
